# Flexible Spiking CPGs for Online Manipulation During Hexapod Walking

**DOI:** 10.3389/fnbot.2020.00041

**Published:** 2020-06-26

**Authors:** Beck Strohmer, Poramate Manoonpong, Leon Bonde Larsen

**Affiliations:** SDU Biorobotics, Maersk McKinney Moller Institute, University of Southern Denmark, Odense, Denmark

**Keywords:** CPG (central pattern generator), spiking neural network (SNN), hexapod robot, biological neuron, neuromorphic engineering

## Abstract

Neural signals for locomotion are influenced both by the neural network architecture and sensory inputs coordinating and adapting the gait to the environment. Adaptation relies on the ability to change amplitude, frequency, and phase of the signals within the sensorimotor loop in response to external stimuli. However, in order to experiment with closed-loop control, we first need a better understanding of the dynamics of the system and how adaptation works. Based on insights from biology, we developed a spiking neural network capable of continuously changing amplitude, frequency, and phase online. The resulting network is deployed on a hexapod robot in order to observe the walking behavior. The morphology and parameters of the network results in a tripod gait, demonstrating that a design without afferent feedback is sufficient to maintain a stable gait. This is comparable to results from biology showing that deafferented samples exhibit a tripod-like gait and adds to the evidence for a meaningful role of network topology in locomotion. Further, this work enables research into the role of sensory feedback and high-level control signals in the adaptation of gait types. A better understanding of the neural control of locomotion relates back to biology where it can provide evidence for theories that are currently not testable on live insects.

## 1. Introduction

In nature, oscillating networks called central pattern generators (CPGs) are known to drive repetitive behaviors like locomotion and breathing in the absence of sensory feedback (Marder and Bucher, 2001). However, in order to adapt locomotive behaviors such as walking patterns, environmental interaction is required to inform the frequency, amplitude, and phase of leg movements (Bidaye et al., 2017). Tripod-like gaits have been observed in deafferented samples but it is still unknown how large a role network topology plays as compared to sensory feedback when it comes to these adaptations (Mantziaris et al., 2017). Recent research on the locust, indicates a balance between neurological network topology and sensory feedback (Reches et al., 2019). This balance differs depending on the insect, with stick insects on the feedback-driven end of the spectrum as compared to the more network-driven cockroach and the locust somewhere in the middle (Reches et al., 2019). Yeldesbay and Daun (2020) study deafferented samples from the stick insect to develop a theory of which neural architectures are most likely to be found in these insects, specifically focusing on the levator-depressor muscle pair. Similar to the deafferented experiments, this study investigates biologically-plausible oscillators without sensory feedback to examine the effects of network topology on walking behaviors.

Neural networks can replicate natural locomotion patterns and adaptations on robots. Most of the current research explores artificial neural networks (ANNs) based on non-spiking neurons but there is a growing body of research into spiking neural networks (SNNs) (Bing et al., 2018). Non-spiking neural networks represent activation as a number to mimic the spike rate of a neuron population whereas spiking neurons represent activation as temporal events, communicating through action potentials called spikes. Importantly, spiking neuron models are able to incorporate time (Walter et al., 2016) because biological studies indicate that neurons communicate through frequency of spikes as well as precise spike timing (Bohte, 2004). Spiking neuron models have also been shown to use fewer neurons for modeling some computations (Maass, 1997). Furthermore, spiking neurons are able to produce bursting behavior which may be important for biological fidelity as a single neural network can initiate different muscle behaviors by changing output bursting patterns (Diehl et al., 2013). Additionally, implementing spiking neurons for deep learning has been shown to decrease power consumption when performing classification tasks, though it comes at the cost of additional delays (Han et al., 2016). Overall, biological plausibility, energy efficiency, increased speed and computational power, and spatio-temporal processing capabilities make spiking neurons desirable for researching biologically-inspired locomotion networks (Bing et al., 2018).

A review of adaptive inter-limb control networks using ANNs was completed by Aoi et al. (2017) focusing on the importance of environmental feedback for coordination. Other works such as Owaki et al. (2013) and Owaki et al. (2017) look into decentralized inter-leg coordination driven by environmental interaction without neural connectivity between legs. These studies confirm adaptability during walking on a quadruped and a hexapod robot, respectively. Similarly, Sun et al. (2018) looks into adaptive self-organized locomotion showing that decoupled non-spiking CPGs (nCPGs) each controlling a single leg can use sensory feedback to learn biologically-plausible quadruped gaits. Self-organizing behavior and adaptability are also shown by Barikhan et al. (2014) using decoupled nCPGs and sensory feedback where robots were able to roll a ball and mimic front leg stepping behavior of insects. These works explore the importance of environmental interaction on coordination using non-spiking neurons. As Aoi et al. (2017) point out, “Legged robots are becoming a valuable tool for understanding the locomotion mechanism including interlimb coordination. In the future,[.] it will be important to enhance biological plausibility and feasibility by the integration with sophisticated models of neural and musculoskeletal systems[.].” This study addresses the need for more biological neural dynamics by using spiking neurons. We explore the effects of decentralized spiking CPGs (sCPGs) without feedback as a primitive control network to gain understanding in a simplistic manner before introducing sensory inputs.

There is a significant body of research into learning using non-spiking neurons which investigates closed-loop feedback control and online adaptation of legged robot locomotion. Thor and Manoonpong (2019) and Pitchai et al. (2019) use learning to find efficient parameters within nCPGs. The controllers are able to adapt amplitude, frequency, and phase of nCPGs within the network. The manipulation of these characteristics can be used to change walking speed (Thor and Manoonpong, 2019), traverse obstacles (Goldschmidt et al., 2012), or compensate for loss of leg functionality (Ren et al., 2015). The mentioned studies use non-spiking neurons, working with traditional ANNs to execute adaptive walking patterns. Building upon the ideas and results from these studies, this work manipulates amplitude, frequency, and phase of sCPGs.

A thorough treatment of sCPG controllers can be found in Gutierrez-Galan et al. (2020) though more focused on engineering solutions than biological. Most notably, Gutierrez-Galan et al. (2020) and Rostro-Gonzalez et al. (2015) have implemented sCPGs capable of online switching between three discrete gaits. These correspond to the three main gaits observed during insect walking—wave gait (Hughes, 1952), tetrapod coordination, and tripod coordination (Graham, 1972) reflecting slow, medium, and fast walking speeds, respectively.

Although some studies using SNNs for robot locomotion have been inspired by CPGs, they mainly focus on the engineering task of getting the robot to walk and less on studying the CPG. As such, Gutierrez-Galan et al. (2020) determine the gait by the network topology, using the spiking neurons to produce a regular frequency which drives the selection of the gait. Rostro-Gonzalez et al. (2015) implement an architecture where a single neuron controls each motor and connections are changed discretely between these neurons to produce the desired behavior. In this way, these studies are able to successfully produce a signal with a steady frequency to produce distinct walking gaits but stray from the known biology. ANNs using non-spiking neurons such as Walknet (Schilling et al., 2013) adopt the switching principle as well. Walknet uses a decentralized structure to control 18 degrees of freedom of a hexapod robot. The controller selects the best fit for the current conditions in order to adapt walking patterns using synaptic weights previously tuned offline. These works switch between gaits using pre-defined network parameters to achieve each pattern. However, biological studies indicate that insects change gaits continuously (Dürr et al., 2018). Therefore, this study expands upon the previous work by implementing a controller capable of online changes as a first step toward a controller that can continuously switch between gaits once feedback is added. Instead of producing a spike train with a regular frequency, this study uses the intrinsic properties of spiking neurons to produce regular bursting which is filtered to create an analog output for the motors.

The aim of this paper is to explore how the network structure can generate coordinated walking behavior. The primary contribution of this research is the implementation of a distributed network of online-adjustable coupled sCPGs which results in a coordinated walking behavior. This research takes the first step toward a real-time adaptable spiking motor control network. Inspired by the biological approach of studying deafferented neural networks, we explore the limitations of an sCPG network when lacking sensory feedback.

## 2. Methods

Biological central pattern generators (bCPGs) are used in nature to generate repetitive behaviors including locomotion. There are two main methods which have been observed for creating rhythmic output. Some networks are driven by “pacemaker neurons” while others create oscillatory output through recurrent connections between non-intrinsically rhythmic neurons (commonly called half-center oscillators). The pacemaker driven networks can be composed of one or several neurons driving oscillation. They are typically pre-motor interneurons which drive the motor neurons. The motor neurons are able to shape the behavior of the bCPG even if they do not contribute directly to the rhythmic output. Neuromodulators also contribute to the updating of bCPGs, changing synaptic strength as well as intrinsic cell properties to affect frequency and phase (Marder and Bucher, 2001).

**Figure 1:**
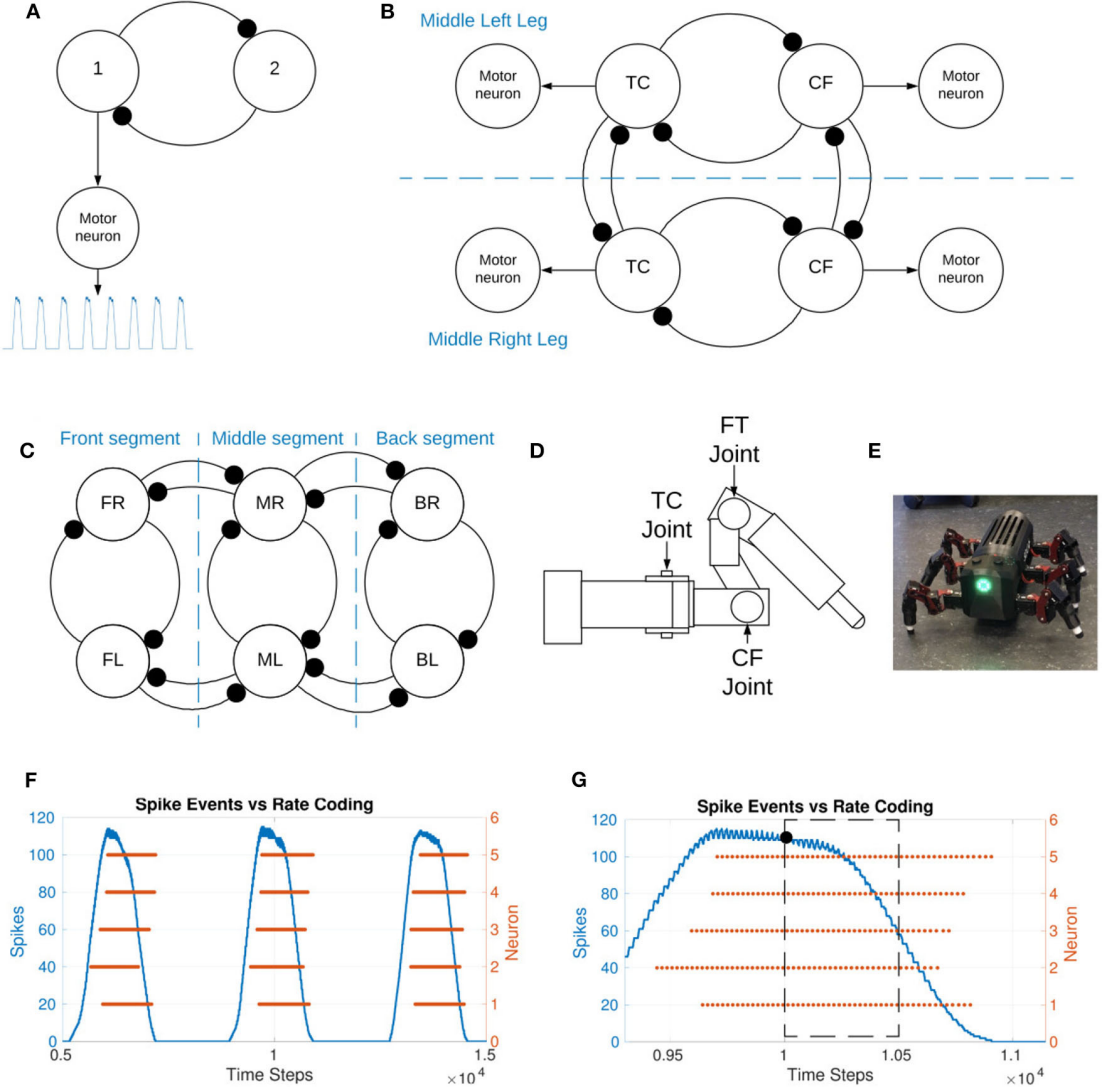
**(A)** Representation of the spiking CPG implementation, 1 and 2 represent the two neuron populations within the sCPG. The motor neuron population is excited by the “first” population within each sCPG. The motor neuron population post-processes the output before relaying it to the joint. **(B)** Representation of the coupling between two legs as well as intra-leg coupling. TC represents the thoraxcoxa joint and CF represents the coxa-femur joint. Each of these consists of a mutually inhibitory pair of neuron populations but is represented by a single circle for figure simplicity. The motor neuron populations are driven by excitatory connections from one of the neuron populations, as seen in **(A)**. The FT, femur-tibia, joint is held fixed. **(C)** Representation of the coupling between the six legs both intra- and inter-segment. Each leg representation consists of two coupled sCPGs as shown in **(B)**. FL, front left; ML, middle left; BL, back left; FR, front right; MR, middle right; BR, back right. The segments are named for clarity. **(D)** Illustration of a single leg of the MORF (Thor, 2019), identifying the location of the movable joints. **(E)** Picture of the hexapod robot used in this study. **(F)** Spike events vs. rate-coding output of a single population consisting of 5 neurons. Spike events are added based on a sliding time window of 5 ms. **(G)** Zoomed in selection of plot F to visualize individual spike events. The number of spikes inside the time window are counted, the resulting value is indicated using a black dot. This shows how spike events translate into an analog output. The simulation data allows the rate-coding algorithm to look forward in time but live experiments would use past data.

Figure 1A shows the generic structure of the sCPGs used in this study with mutually inhibitory neuron populations sending oscillatory output to a motor neuron population. The sCPGs implemented in this study are designed as pacemaker networks, taking advantage of neuron parameters that allow regular bursting. As the neurons are intrinsically firing, excitation is created through a static current bias together with gaussian white noise.

Each pacemaker network consists of two neuron populations mutually inhibiting each other. This configuration mimics the biological setup of two coupled neuron populations for controlling an antagonistic muscle pair (Bidaye et al., 2017).

Initial testing of the network began with just one neuron per population, inspired by the sea slug Dendronotus iris. It has one of the smallest networks found in nature consisting of two half-center oscillators, a total of four neurons, generating anti-phasic motor outputs for swimming (Sakurai and Katz, 2016). Once the output is confirmed, the population sizes are increased until spiking stabilized from all motor neuron populations. Based on this testing, each neuron population consists of five neurons. Results per population size can be found in the Supplementary Material.

### 2.1. Neuron Model

The adaptive exponential integrate and fire (AdEx) neuron model is selected for the network because it is able to model different neuron firing patterns, including bursting behaviors, while reducing computational complexity (Brette and Gerstner, 2005). The Izhikevich neuron model is also able to produce bursting behaviors (Izhikevich, 2003) and we expect the network behavior could be reproduced using Izhikevich neurons.

Equations (1) and (2) define the dynamics of the adaptive exponential integrate and fire neuron model.

(1) $$\begin{array}{l} C\frac{dV}{dt}=-g_{L}\left(V-E_{L}\right)+g_{L}\left(\Delta T\right)e^{\frac{V-V_{T}}{\Delta T}}-w+I_{e} \end{array}$$

when *V* > *V*_*T*_ then *V* → *V*_*r*_

(2) $$\begin{array}{l} \tau_{w}\frac{dw}{dt}=a\left(V-E_{L}\right)-w \end{array}$$

when *V* > *V*_*T*_ then *w* → *w* + *b*

where C is membrane capacitance, *V* is membrane voltage, *E*_*L*_ is resting potential, *g*_*L*_ is leakage conductance, *I*_*e*_ is bias current, *a* is sub-threshold adaptation conductance, *b* is spike-triggered adaptation, Δ_*T*_ is slope/sharpness factor, τ_*w*_ is adaptation time constant, *V*_*T*_ is spike threshold, and *w* is spike adaptation current.

In Equation (1), the first term is the leakage while the second term is the exponential non-linearity defining sharpness. At the reset threshold, 'V' is reset to a fixed variable (*V*_*r*_) whereas ‘w' is reset by a fixed amount (b) allowing it to accumulate during the spike train and enabling bursting behaviors (Naud et al., 2008).

The values to be used are extracted from the “regular bursting” parameters as defined by Naud et al. (2008). However, three parameters are manually tuned through trial and error: *V*_*T*_, *I*_*e*_, and *t*_*ref*_. The paper does not define a refractory period so a biologically-plausible refractory period of 2 ms is selected (Roeder, 1998). Once this is established, *I*_*e*_ can be determined by using the original (Naud et al., 2008) parameters and increasing the bias current incrementally. Finally, the range of *V*_*T*_ is tested by stepping through a range and finding the minimum and maximum values. During these trials, the resulting parameters are considered usable when they result in stable oscillatory behavior within a desirable frequency range.

Table 1 outlines the values used for the adaptive exponential integrate and fire neuron model vs. the values from the Naud et al. (2008) paper. The shape of the sCPG is constructed using the regular bursting behavior of the neurons. However, the current is increased to promote repetitive spiking (Naud et al., 2008) and gaussian white noise is added to mimic noise in the nervous system (Faisal et al., 2008). The mean and standard deviation of the noise is manually found through testing as investigation into these values is outside the scope of this work.

**Table 1:** Parameter comparison for adaptive exponential integrate and fire neurons Equations (1) and (2).

**Parameter name**	**Symbol**	**Naud et al. (2008)**	**Actual value**
**Neuron model parameters**			
Capacitance	*C*	200 pA	200 pA
Leakage conductance	*g*_*L*_	10 ns	10 ns
Resting potential	*E*_*L*_	−58 mV	−58 mV
Spike threshold	*V*_*T*_	−50 mV	Varied
Slope factor	Δ*T*	2 mV	2 mV
Adaptation time constant	τ_*w*_	120 ms	120 ms
Sub-threshold adaptation conductance	*a*	2 ns	2 ns
Spike-triggered adaptation	*b*	100 pA	100 pA
Reset voltage	*V*_*r*_	−46 mV	−46 mV
Bias current	*I*_*e*_	210 pA	500 pA
Refractory period	*t*_*ref*_	0 ms	2 ms

The second ordinary differential equation allows for the possibility of natural periodic behavior stemming from the complex dynamics of a two-dimensional system. For this research, the voltage dependent conductance-based model of neuron dynamics (Equation 1) is used together with an alpha synaptic function (Equation 3). A current-based model was also tested but the same oscillations could not be achieved so it was not implemented.

(3) $$\begin{array}{l} g_{syn}\left(t\right)=g_{syn}\alpha^{2}te^{-\alpha t} \end{array}$$

This function describes the change in conductance after a spike, allowing current to flow along the synapse. It includes a term for synaptic rise time (α) and the strength of the synapse (*g*), both terms influence how often a neuron spikes (Van Vreeswijk et al., 1994).

The neuron and synapse models have been chosen to allow for intrinsically bursting, online-adaptable periodic output. This creates an output reminiscent of a sinusoidal once a low pass filter is applied. The AdEx neuron model consists of two differential equations creating a two dimensional system. Two-dimensional systems have closed orbits allowing for periodic behavior to emerge, making them well-suited for driving oscillations.

### 2.2. Network Topology

The implemented sCPG network architecture is biologically-inspired by the stick insect, using one sCPG for each of the joints. The three main leg joints identified in stick insects are the thorax-coxa (TC), coxa-trochanter (coxa-femur) (CF), and femur-tibia (FT) (see Figure 1D for location of these joints on the robot leg). These are responsible for horizontal, vertical, and extension movements respectively (Bidaye et al., 2017). The individual sCPGs are coupled to the other joints within the leg and between the legs in order to create a distributed network of oscillators.

Figure 1B is a high-level overview of the coupling between two legs as well as within a single leg. Two joints per leg are implemented and coupled to their contralateral counterpart to form the inter-leg connection. In order to meaningfully incorporate the FT joint, the load sensory feedback must be provided as inhibitory feedback to the “flexor” neuron of the bCPG. This allows the “extensor” neuron to hold the stance phase longer so that the insect does not enter swing phase when there is a substantial load on the leg (Fukuoka et al., 2015). The FT joint is held fixed in this study due to the lack of sensory feedback.

The joint coupling is weak, varying in a range from −0.5 to −1.0 nS while the sCPG, inter-leg, and inter-segment coupling is a stronger coupling of −10 nS. Inter-segment coupling is shown in Figure 1C, it also consists of a mutual inhibitory connection between each joint and its neighboring ipsilateral counterpart. The complete network diagram with all neuron populations and connections can be found in the Supplementary Material.

The coupling of legs and joints to their immediate neighbors is based on observations in nature where sensory feedback to a single leg affects those closest to it (Mantziaris et al., 2017). The design of the coupled network allows for frequency, amplitude, and phase to be updated while running, relying on inter-leg and inter-segment coupling to coordinate leg movements.

### 2.3. Manipulation of Network Characteristics

The scaling parameter, *V*_*T*_, is used to update the inter-burst interval frequency of the oscillators. *V*_*T*_ offsets the membrane potential as it defines the minimum of the V-nullcline (Naud et al., 2008) thereby affecting frequency.

**Figure 2:**
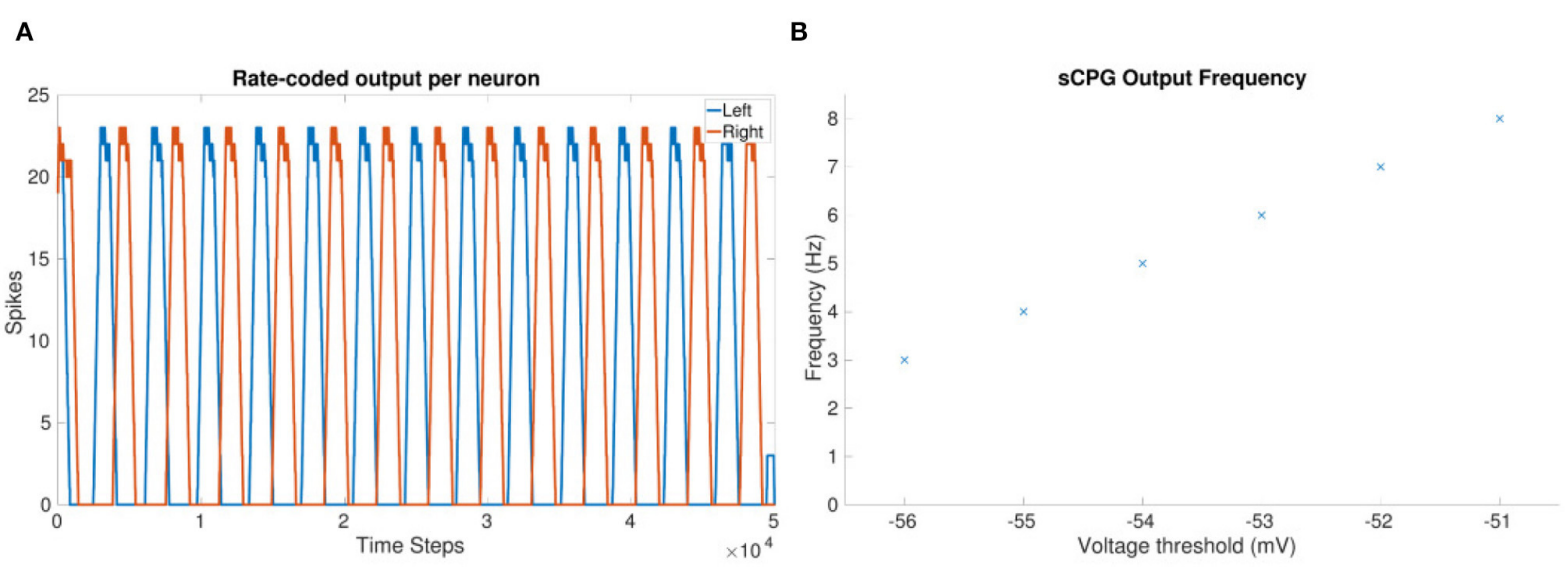
**(A)** Rate-coded sCPG output from each neuron, left and right, when using only two neurons in total. *V*_*T*_ = −56 mV. Rate-coding mimics the role of a muscle which acts as a low pass filter (Hooper et al., 2007). **(B)** Observed frequency at each voltage threshold value using 1 mV steps.

As the frequency increases, the amount of spikes per burst decreases. Therefore, the coupling weight between joints must be increased to compensate for the reduction in spiking. A desirable coupling weight is found through testing of the network. In the equation, frequency is represented by *V*_*T*_ which has a linear relationship to frequency, as shown in Figure 2B.

(4) $$\begin{array}{l} w_{coupling}=\left(V_{T\text{\_}min}-V_{T}\right)0.1-1 \end{array}$$

*V*_*T*_*min*_ is the minimum threshold value discovered to give meaningful results from the implemented network topology. Equation (4) is derived through testing, results showing frequency adaptation and the affect on joint coupling can be found in section 3.

The amplitude (magnitude) of muscle activation during stepping is usually updated in response to sensory feedback (Schmitz et al., 2015). Biological research suggests that rhythmic motor patterns are likely affected by short-term plasticity (McDonnell and Graham, 2017). This is mimicked artificially here by changing the synaptic weight. Due to the open-loop nature of the network, the change in amplitude is prompted by updating the excitatory weights to the motor neuron populations by a set value. Once sensory feedback and synaptic learning are incorporated into the network, this will replace the manually selected weight values.

The phase shift between sCPGs relies on the ability to know the frequency of the individual sCPGs. Furthermore, they must run at the same frequency or at a whole number multiple relative to each other in order to create a meaningful phase-lock. A whole number ratio relating the period of each leg's stepping cycle has also been observed when studying walking in stick insects (Foth and Bässler, 1985). The inter-leg and inter-segment phase shift is a consequence of the coupling between oscillators. However, the interjoint phase shift of 90° between the body-coxa and coxa-femur joints is chosen based on the requirements of the physical robot used for validation (Thor, 2019). The delay to create a 90° phase shift between the TC and CF joints in a single leg is calculated using the output frequency of the sCPG as shown in Equation (5).

(5) $$\begin{array}{l} delay=\frac{1}{2\left(frequency-1\right)}1000 \end{array}$$

Using the delay calculation, when outputting a frequency of 3*Hz*, a delay of 250*ms* will be applied. Note, this is theoretically a 90°phase shift for 2*Hz* (period = 500*ms*⇒ half period = 250*ms*), manual adjustment of the equation is found through testing.

### 2.4. Experimentation

Simulation with NEST (Jordan et al., 2019) on a PC and validation on a physical robot are used to confirm the sCPG network design. The sCPG output plots use rate coding to plot spikes in time bins of finite amounts. The filter is implemented as a sliding time window. This is comparable to the muscles in biology which act as low pass filters, filtering variations in spike timing (Hooper et al., 2007). Figures 1F,G show how spike events are added in order to provide an analog output for the motors. The original code used to create the plots in section 3 is available on GitLab (Strohmer, 2020). Initially, a simple two neuron sCPG is simulated in order to confirm spiking and coupling behaviors using the neuron parameters defined in Table 1 before moving onto the full network simulation.

Manual testing revealed that the voltage threshold must be constrained within a defined range from −56 to −51 mV when using a current bias of 500 pA. A linear relationship between frequency and spike threshold is observed when using the defined parameters.

Real-time adaptation of amplitude is normally governed by sensory feedback. For testing, the excitatory synapse to each motor neuron population is increased exponentially by a factor of three to show that amplitude can be updated while running. The excitatory synaptic weight starts at 2 nS, ending at 486 nS after the six iterations defined by the voltage threshold range. The weight range is determined through trial and error.

The delay between joints must be adapted when the frequency changes in order to keep the phase. The delay is calculated by Equation (5), ranging from 71 to 250 ms based on attainable frequencies. It is updated each time the voltage threshold changes. The delay is placed on the excitatory synaptic connection from the coxa-femur joint to the corresponding motor neurons.

A validation test is performed on the Modular Robot Framework (MORF) (Thor, 2019) shown in Figure 1E. It is both a simulated robot in V-REP (Rohmer, 2013) and a physical hexapod robot with 18 degrees freedom. Each leg has three movable joints corresponding to the TC, CF, and FT joints as seen in Figure 1D. The MORF is fitted with an Intel NUC which runs as a ROS node to send and receive data wirelessly from a PC. The values produced in the NEST simulation by the sCPG network are exported to comma delimited files, one per joint. This data is used to send position values to the relevant joint on the robot at a rate of 60 Hz as required (Thor, 2019). A python script translates the original sCPG output values to radians and enforces an upper and lower bound to ensure the motors operate within their designed range of motion. The python script is available on GitLab (Strohmer, 2020), it handles reading and writing to the robot through a robot operating system (ROS) interface. The network is first simulated with *V*_*T*_ = −56*mV* and again when updating the threshold from *V*_*T*_ = −55 to −54*mV*. The joint positions for all 12 joints to be controlled are recorded for each NEST simulation. These values are tested on the simulated MORF in V-REP before confirming locomotion on the physical robot.

Torque measurements are recorded from the Dynamixel motors during robot locomotion in order to plot the swing and stance phase of each leg. The torque feedback is actually a current measurement converted to torque using the “torque to current value ratio” defined by Dynamixel (ROBOTIS, 2018). The load of the robot is mostly divided between the CF and FT joint during walking as can be intuited from Figure 1D. Therefore, the torque values plotted are the sum of the absolute values of these two joints. As torque is the rotational force measurement, negative and positive only indicate a direction so plotting the absolute values is valid.

The experiments performed on the MORF are offline as the position values sent to the motors are pre-recorded data obtained from the NEST simulations. However, the NEST simulations are considered to be online manipulations as they calculate updated network parameters and change neural characteristics while running. This is achieved by dividing the simulation into smaller time intervals of one second each and updating necessary information between each interval. All rate-coded plots are based on NEST simulation output data. The torque measurements are recorded during MORF walking and the gait diagram is based on data recorded during V-REP simulation of the MORF walking.

## 3. Results and Discussion

The output of a two neuron sCPG shown in Figure 2A confirms spiking and anti-phasic outputs due to mutual inhibitory coupling.

This result indicates that the neuron parameters producing regular bursting can be used to create an sCPG in the style of a pacemaker network. When expanding the network to six legs consisting of two joints each, using a single neuron per population is no longer viable. It does not allow for all spikes to reach the motor neurons controlling the CF joints of the middle or back legs. Further simulations (see Supplementary Material) reveal that a minimum of five neurons per population yield a more reliable result.

**Figure 3:**
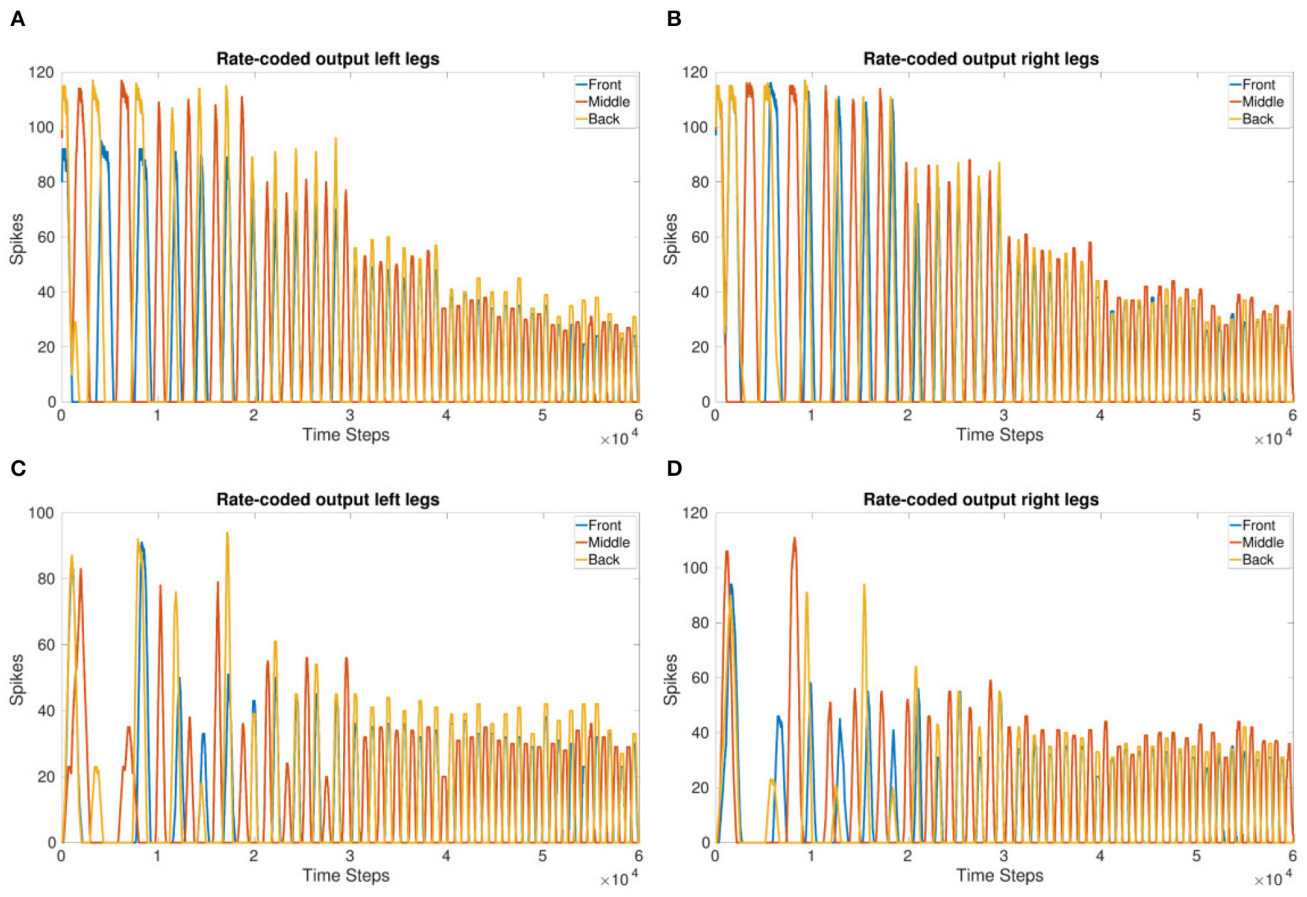
**(A,B)** Rate-coded output of the left legs **(A)** and right legs **(B)** updating *V*_*T*_ every 1 s (10,000 time steps). *V*_*T*_ = −56 to −51 mV. Front and back legs on the same side spike together indicating a tripod gait. **(C,D)** Amplitude of the rate-coded output is updated by increasing the excitatory synaptic weight exponentially, multiplying the weight by 3 for each increase in *V*_*T*_ occurring every 1 s. This shows the amplitude can be managed while the system is running.

Figures 3A,B shows the frequency updating over time, *V*_*T*_ is incremented by 1*mV* every 1 second resulting in an increase in frequency as well as a reduction in the amount of spikes per time bin. Figure 2B shows the observed frequency for each whole value of *V*_*T*_. The smallest voltage threshold shows a frequency of 3 Hz, this increases to 8 Hz for the largest tested value. The frequencies are also observable by counting the peaks in Figures 3A,B.

The results show that the inter-leg coupling holds the phase during the frequency adaptation so that after the initial transients, the front and back legs on the same side move in-phase. This indicates that a stable tripod gait is maintained with the implemented network coupling.

Figures 3C,D show the adjustment of amplitude while the frequency is increasing. This is in contrast to Figures 3A,B where the number of spikes decreases significantly. The 1 s threshold updates are clearly visible in Figures 3A,B whereas they are evened out over the last 3 s in Figures 3C,D.

Even though the starting thresholds produce larger amplitudes, they are still smaller than the initial amplitudes shown in the frequency adaptation plot (Figures 3A,B) indicating that updating the excitatory synaptic weight does change the number of output spikes as expected. Even though the initial transients are more apparent in Figures 3C,D, after settling, the tripod gait returns due to the mutually inhibitory connections between legs.

**Figure 4:**
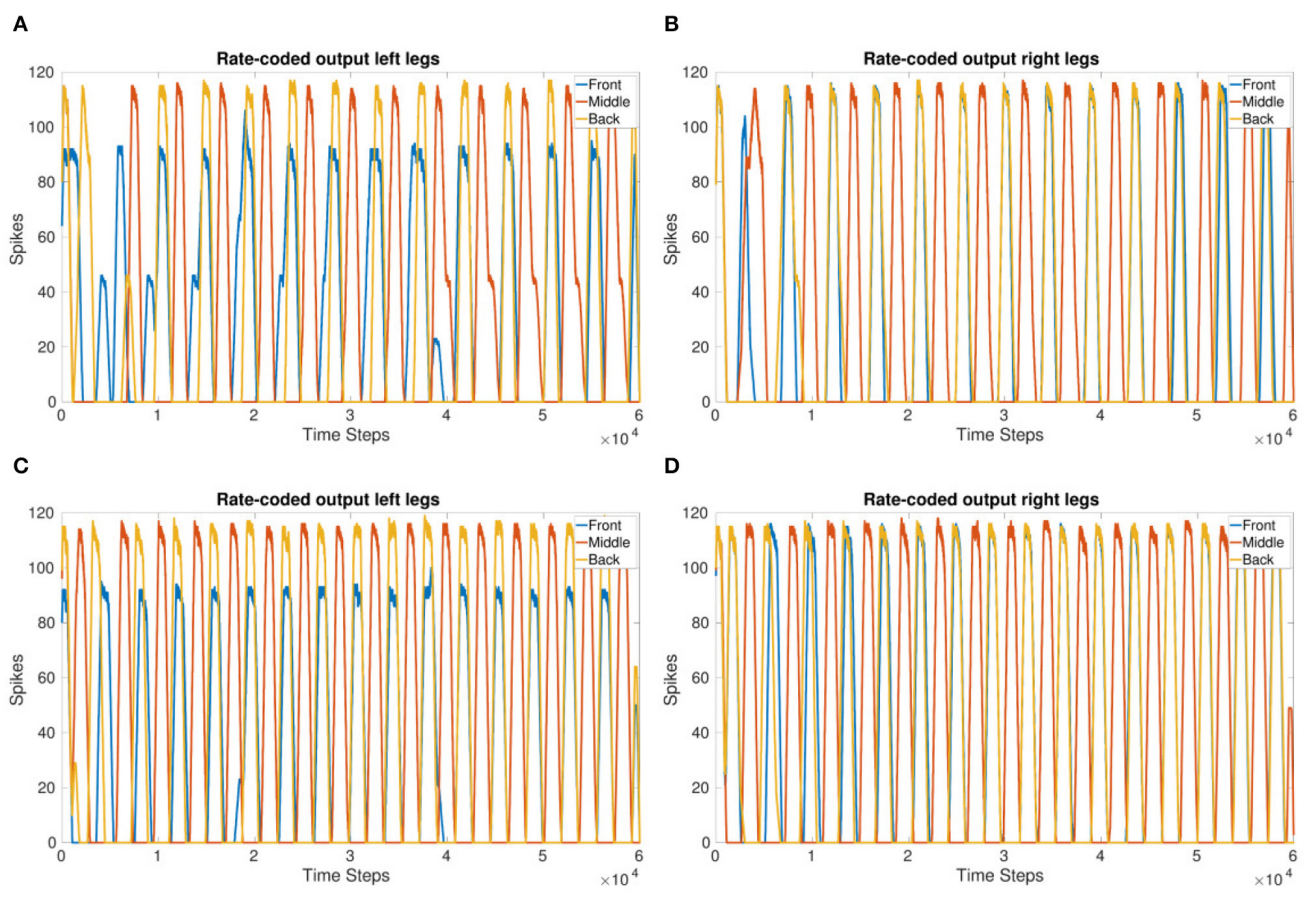
Coupling comparison showing the stability added by fully coupling sCPGs to each other. **(A,B)** Leg coordination when only “first” neuron populations are coupled to their corresponding counterpart in the neighboring sCPG. **(C,D)** Leg coordination when both neuron populations of each sCPG are coupled to their corresponding counterpart in the neighboring sCPG. These simulations indicate that connectivity between sCPGs informs network stability.

Coupling corresponding neurons in sCPGs added stability to inter-leg and inter-segment phase differences. The legs are able to move in a stable, coordinated pattern as can be seen in Figures 4C,D. The single joint coupling shows that the front and back legs do not always move in-phase (Figures 4A,B). However, once coupling between corresponding neuron populations in each sCPG is introduced, the front and back legs move in-phase creating a steady tripod gait. The front left leg does not have the same amplitude because of the sequential nature of the simulation where the front sCPG joint outputs are calculated first combined with the use of the minimal neuron population size of 5. Once the neuron populations are increased to 6, the amplitudes for the front legs are comparable to the other legs. The joint output for each leg when using six neurons is shown in the Supplementary Material.

**Figure 5:**
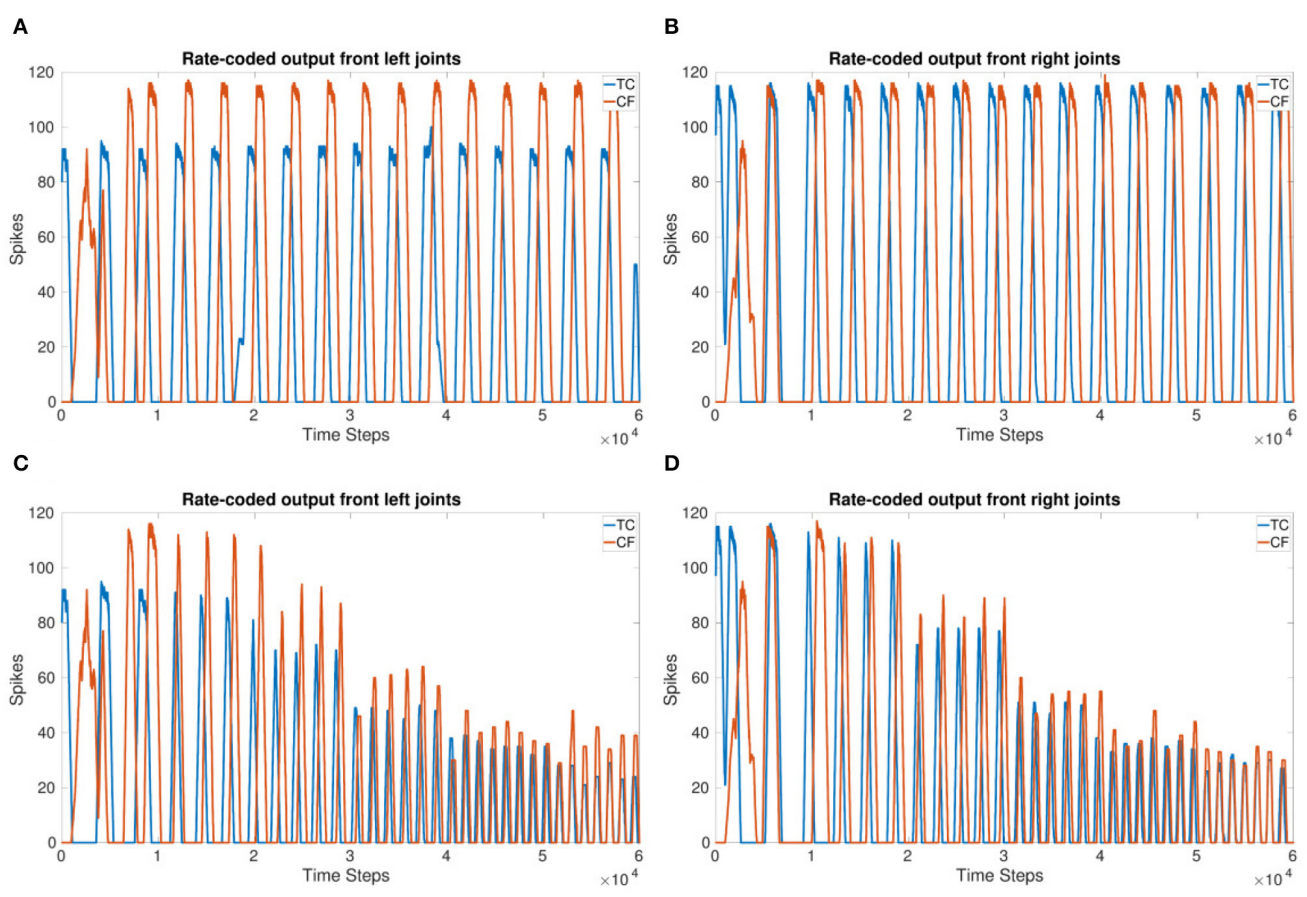
**(A,B)** sCPG output of front left **(A)** and front right **(B)** joints with a 90° phase shift. **(C,D)** sCPG output of front left **(C)** and front right **(D)** joints with a 90° phase shift adapting to frequency. *V*_*T*_ is incremented by 1 mV every 1 s (10,000 time steps). Delay is updated based on *V*_*T*_ using Equation (5).

Figures 5A,B show the 90° phase shift between the TC and CF joints in each of the front legs. When using a delay of 166 ms for 3 Hz, the expected delay required, the outputs of the joints are in-phase. Increasing the delay to 250 ms for 3 Hz produces the desired 90° phase shift. Figures 5C,D confirm the phase is able to adapt to a change in frequency and keep a specified delay. However, for the highest frequency, the phase takes an initial adjustment period where the joints are in-phase before moving toward a 90°phase shift again. This indicates the delay and joint coupling weights are not optimized.

**Figure 6:**
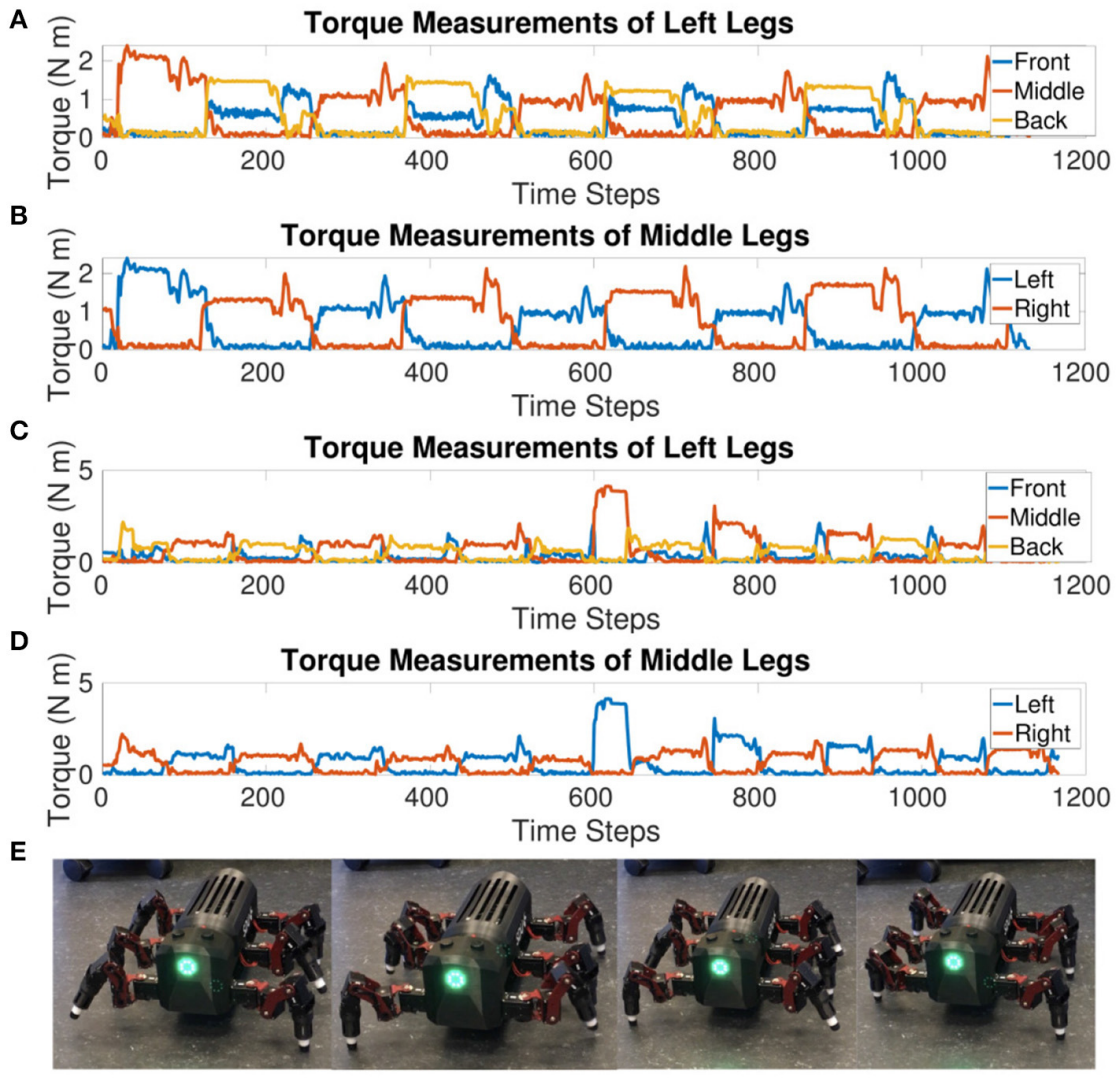
**(A)** Sum of the absolute values of torque measurements from the CF and FT joints for each leg on the left side of the MORF while walking at a single frequency. The front and back legs move together while the middle leg is out of phase compared to both. This shows the tripod gait holds when testing on a physical robot. **(B)** Sum of the absolute values of torque measurements from the CF and FT joints for the middle legs of the MORF while walking at a single frequency. The left and right legs move out of phase, confirming a tripod gait. **(C)** Sum of the absolute values of torque measurements from the CF and FT joints for each leg on the left side of the MORF while switching to a new frequency. After an initial transient, the legs are able to re-adjust and return to a tripod gait. **(D)** Sum of the absolute values of torque measurements from the CF and FT joints for the middle legs of the MORF while switching to a new frequency. The left and right legs move out of phase though the stance phase is visibly shorter for the left leg after the frequency transition. **(E)** Stills taken from the video of the MORF walking.

Figure 6 confirms a tripod gait is maintained when the robot is walking. The front and back legs move in-phase while the middle leg moves out of phase. Likewise, left and right legs within the same segment move out of phase. There are transients visible at the end of the stance phase. The front and back legs seem to compensate for each other during the end of the stance phase where the front leg increases in torque as the back leg decreases before both go to zero during their mutual swing phase. This increase in torque is also visible in the original MORF torque plots (Thor, 2019) indicating that this could be due to the robot's morphology. If the leg slips at the end of the stance phase and then catches, this would show as a decrease in torque followed by a spike as depicted in Figure 6.

The torque value when updating the voltage threshold half-way through from *V*_*T*_ = −55*mV* to −54*mV* shows a tripod gait is maintained after a frequency transition during walking. The transient in the middle of Figures 6C,D pinpoints when the frequency is changing. After the initial transient, the robot settles back into a steady tripod gait. However, the stance phase is shorter for the left middle leg, this is most likely an artifact of the frequency shift and mutual inhibition promoting anti-phasic behavior. The lack of sensory feedback limits the ability to re-adapt to an equal duty cycle for the left and right legs. Instead, the weight of the connections and delay between sCPGs will determine how the joints settle into anti-phasic behavior, eventually defining the length of the swing vs. stance phase for each. This result indicates that the selected weights and delays are not optimal but the network is functional.

**Figure 7:**
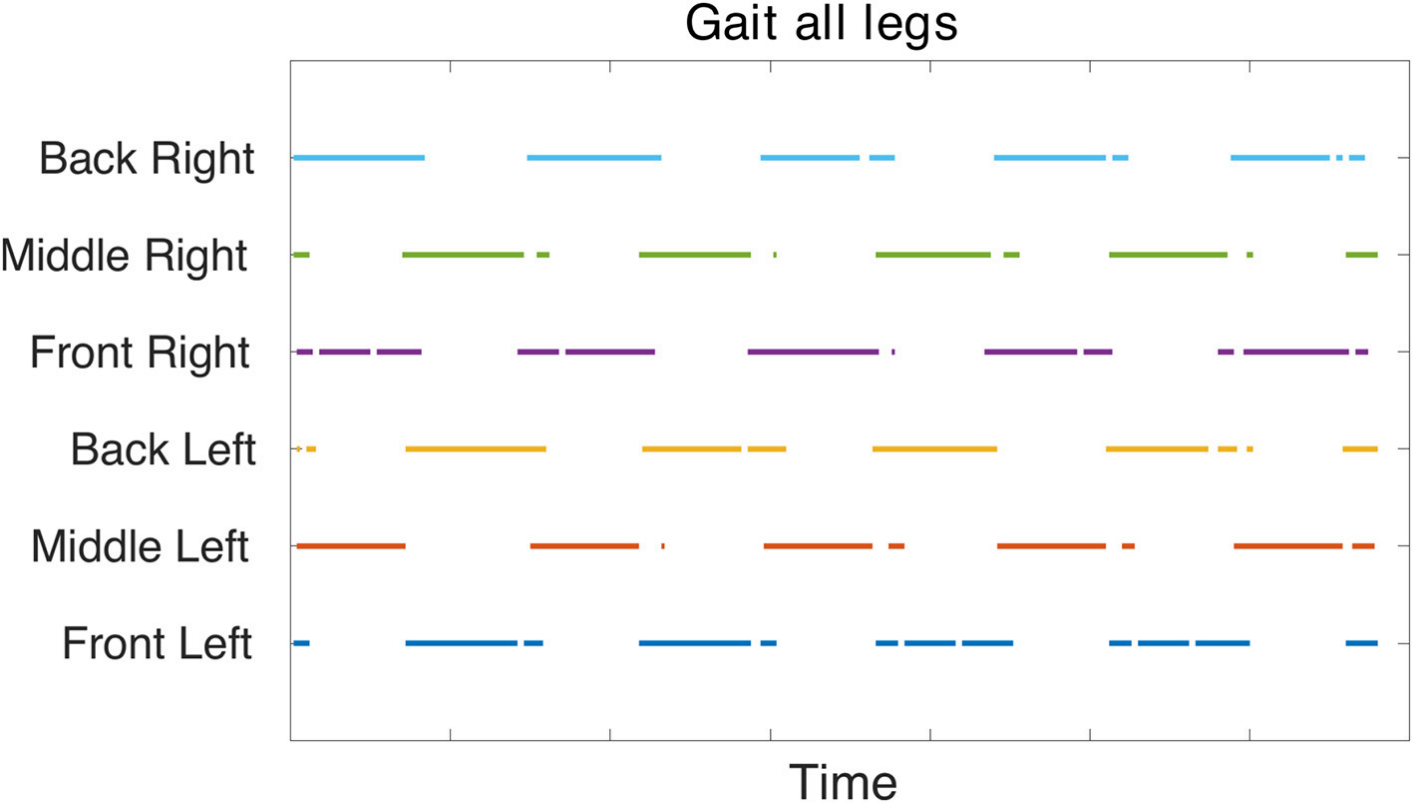
Gait diagram based on force sensor feedback collected during simulation in V-REP. The force measurement from the CF and FT joints are rectified and added together. If this absolute force measurement is above a specific threshold based on the extracted data, it is determined as foot contact with the ground. Therefore, the colored points indicate stance phase and confirm a tripod gait.

The gait diagram (Figure 7) confirms the tripod gait. The overlap of stance phase can be seen. This is due to the slow walking frequency which creates transition phases where all legs are in contact with the ground. The stance phase is occasionally interrupted, this is could be an effect of improper parameter tuning of the FT joint's position.

The remainder of the torque plots for the single and transitioning frequency can be seen in the Supplementary Material. A link to the video of the MORF walking can also be found in the Supplementary Material.

The frequency, phase, and amplitude are confirmed to be interdependent. Changing the frequency of a single joint affects the phase difference in relation to other joints. This is addressed by using the same frequency for all joints at any given time, allowing the mutually inhibitory connections to force anti-phasic behavior in neighboring legs and seems to be a robust solution. However, the 90° phase shift between the intra-leg joints created through a delay to the motor population is more susceptible to frequency changes since it is a calculated parameter rather than a natural product of the network topology. The amplitude is also directly influenced by frequency as less spikes occur per time bin as frequency increases. Increasing the number of neurons in the motor population or increasing connectivity from the sCPG population to adapt to this reduction are potential solutions. However, these inter-dependencies require deeper investigation to construct biologically-plausible network topologies with desired output characteristics.

## 4. Conclusion

This paper introduces a decentralized spiking CPG network inspired by insect neurobiology. The amplitude, frequency, and phase can be manipulated while the network is running indicating that these characteristics can be updated online to further explore the role of sensory feedback in shaping locomotion. A tripod gait is achieved based on a biologically-inspired open-loop network topology indicating that topology does play a part in walking coordination. This research lays the groundwork for further investigation into online adaptable spiking networks and the role of network topology compared to environmental feedback.

Future work should incorporate sensory feedback into the distributed sCPG network. Once this is implemented, the network can begin reacting to its environment. Learning algorithms should be explored to optimize synaptic strength to the motor neurons as well as the coupling weights between the sCPGs. The addition of the third leg joint using load sensory feedback to mechanically couple this joint to the existing network should be investigated. This will allow for a more biologically accurate representation of walking where the leg is extended and flexed during the swing and stance phase (Bidaye et al., 2017). Additionally, sensory feedback could help to adjust phase quicker during frequency adaptations and may also provide stability through mechanical coupling of joints.

The creation of a network capable of online, reactive adaptation to the environment allows for biological hypotheses to be explored in a controlled environment with detailed feedback from sensors and actuators. This could provide more information on the interaction of forces during locomotion since there are limits when working with live insects and deafferented samples. Online adaptation also lends itself to online learning, providing the opportunity to examine stepping patterns, both rhythmic and non-rhythmic. This leads to more robust locomotion and exploration behaviors, allowing robots to navigate rougher terrains.

## Data Availability Statement

Publicly available datasets were analyzed in this study. This data can be found here: https://gitlab.com/esrl/scpg-network-simulation.

## Author Contributions

BS and LL conceived of the presented idea. BS reviewed biology and synthesized the findings to a complete, working sCPG model. BS formulated the theory and performed the computations. The manuscript was written by BS with support from LL and PM. LL supervised the project and provided the funding. All authors contributed to the article and approved the submitted version.

## Conflict of Interest

The authors declare that the research was conducted in the absence of any commercial or financial relationships that could be construed as a potential conflict of interest.
