# Supplementary material for: Flexible Spiking CPGs for Online Manipulation During Hexapod Walking
Source: Front Neurorobot. 2020 Jun 26;14:41. doi: 10.3389/fnbot.2020.00041 (PMC7333644; doi:10.3389/fnbot.2020.00041)
Supplement: Supplementary file 1 [file Data_Sheet_1.PDF]

Supplementary Material

1 SUPPLEMENTARY TABLES AND FIGURES

1.1 Figures

Rate-coded output for various sCPG population sizes

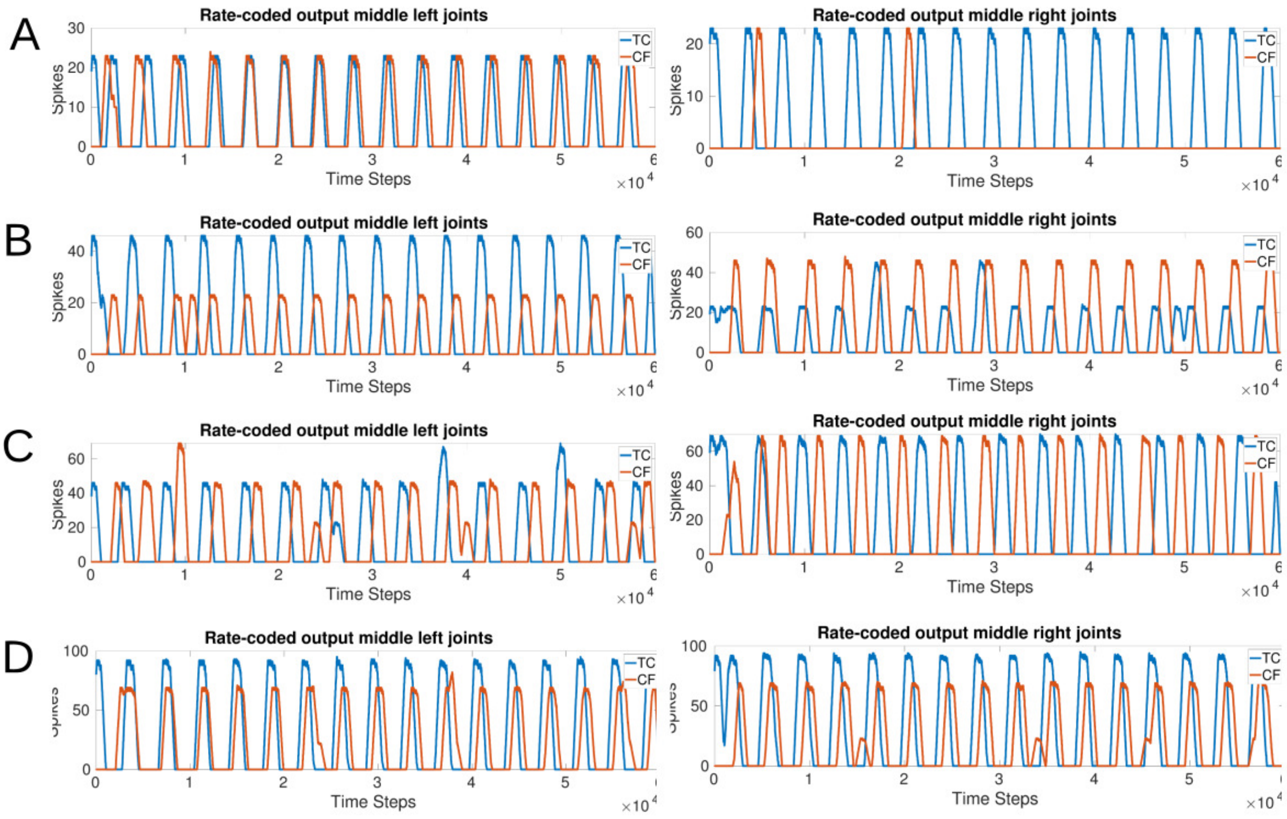

Figure S1. Rate-coded output for increasing population sizes. A: One neuron per population. B: Two neurons per population. C: Three neurons per population. D: Four neurons per population.

Rate-coded output for 6-neuron sCPG population

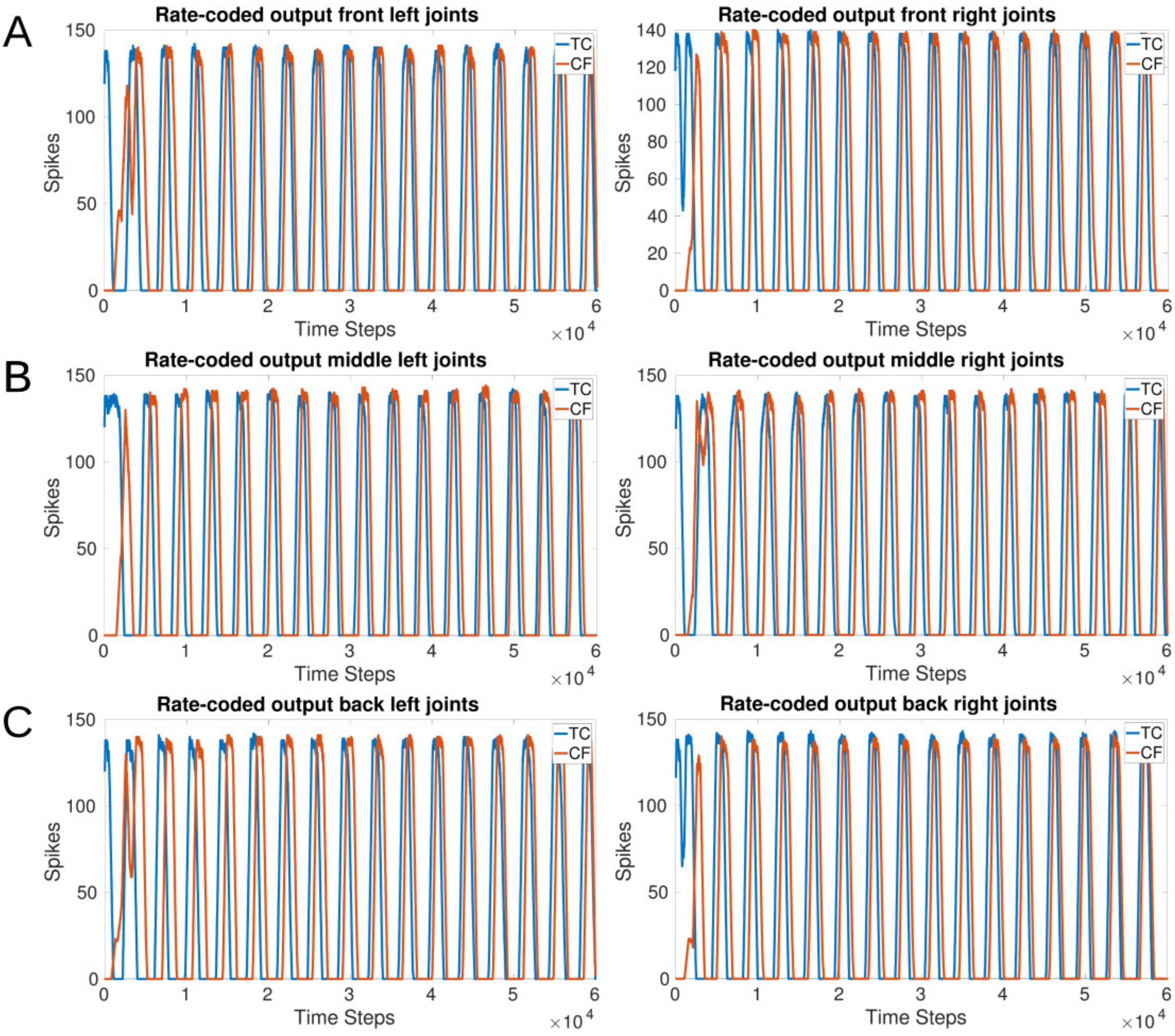

Figure S2. Rate-coded output for sCPGs with 6 neurons per population. A: Front legs. B: Middle legs. C: Back legs.

Video of MORF walking

Torque output for MORF walking at a single frequency

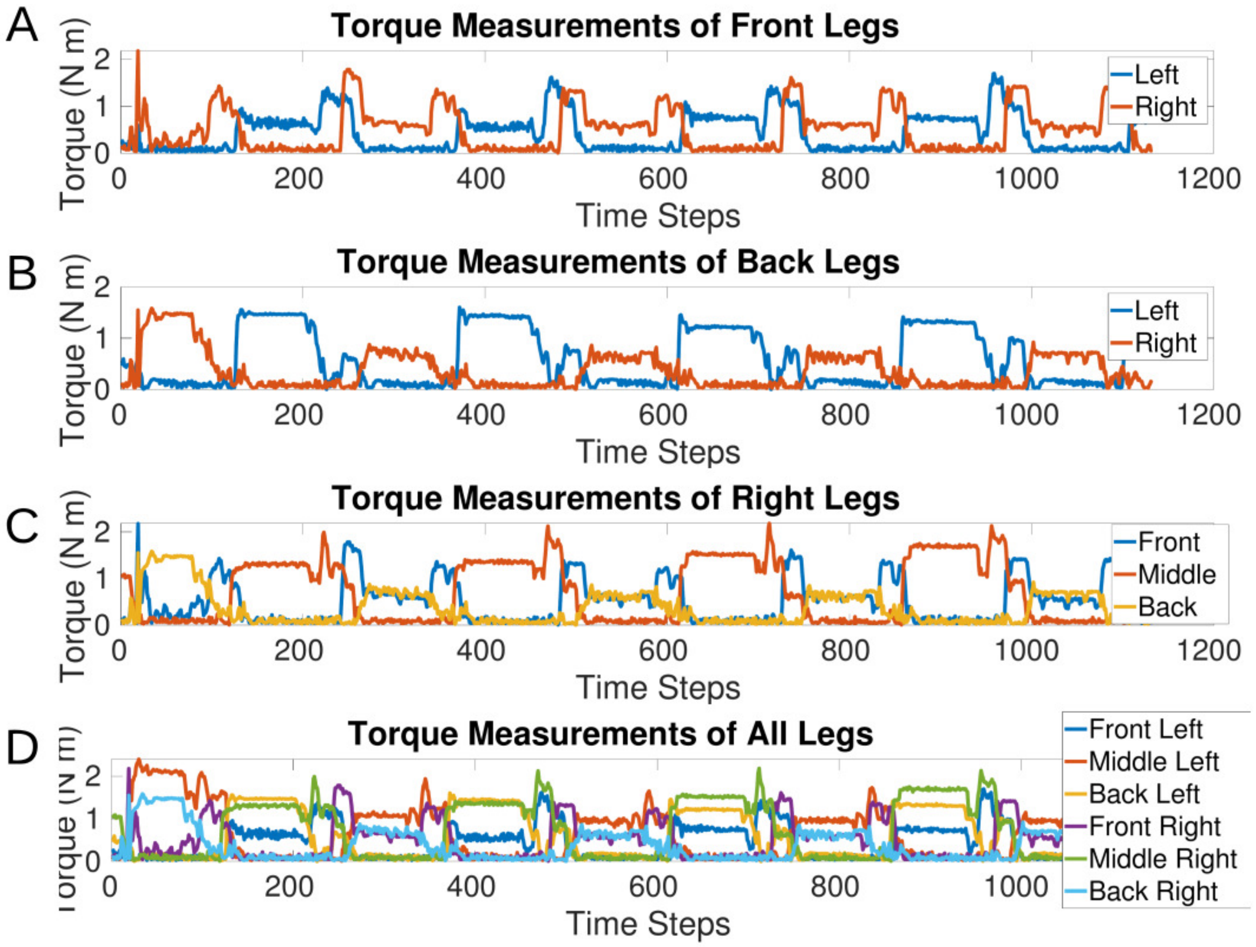

Figure S3. Converted torque measurement from Dynamixel motors when walking at a constant frequency.

Torque output for MORF walking when switching between frequencies

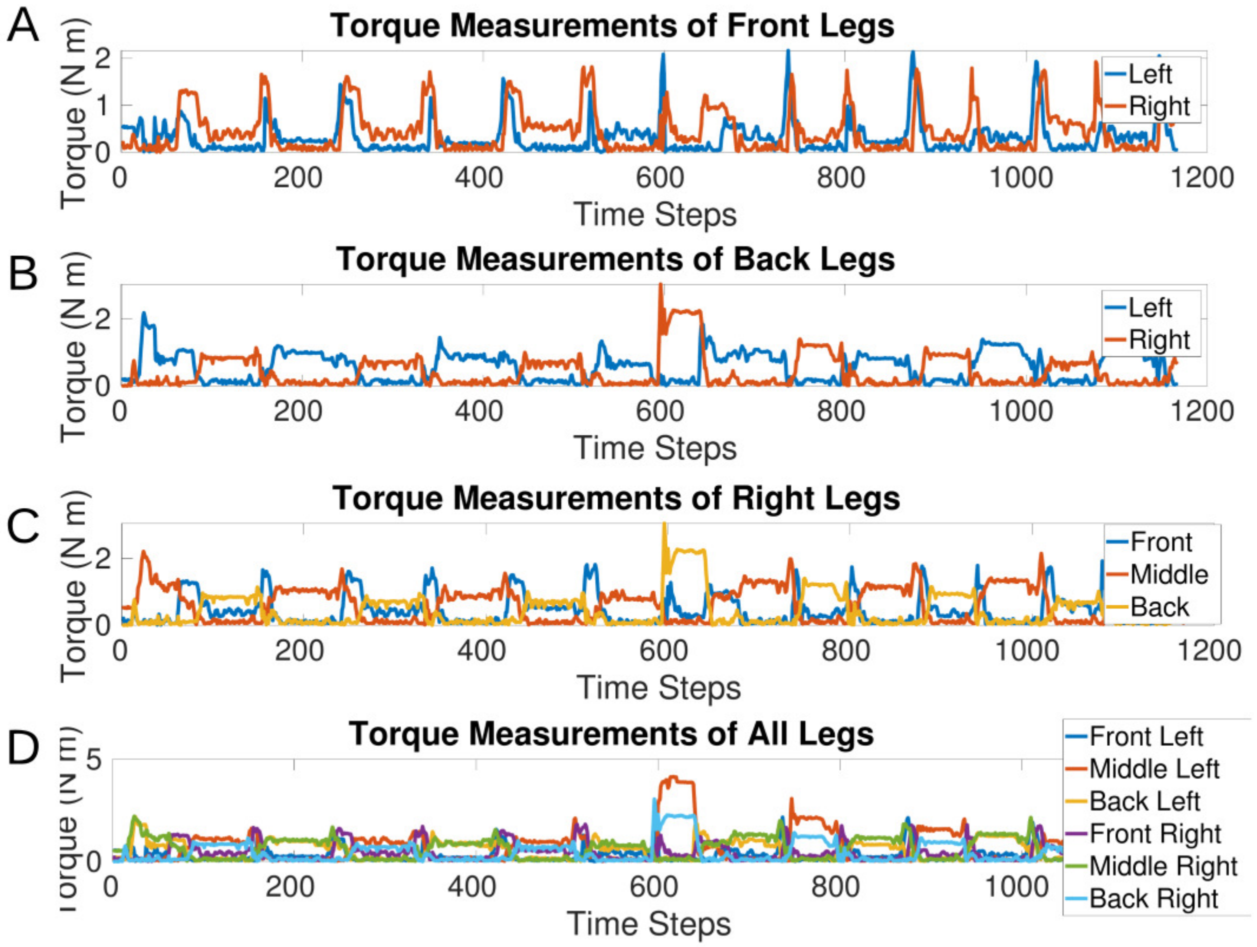

Figure S4. Converted torque measurement from Dynamixel motors when switching from one frequency to another.

Complete network of distributed sCPGs

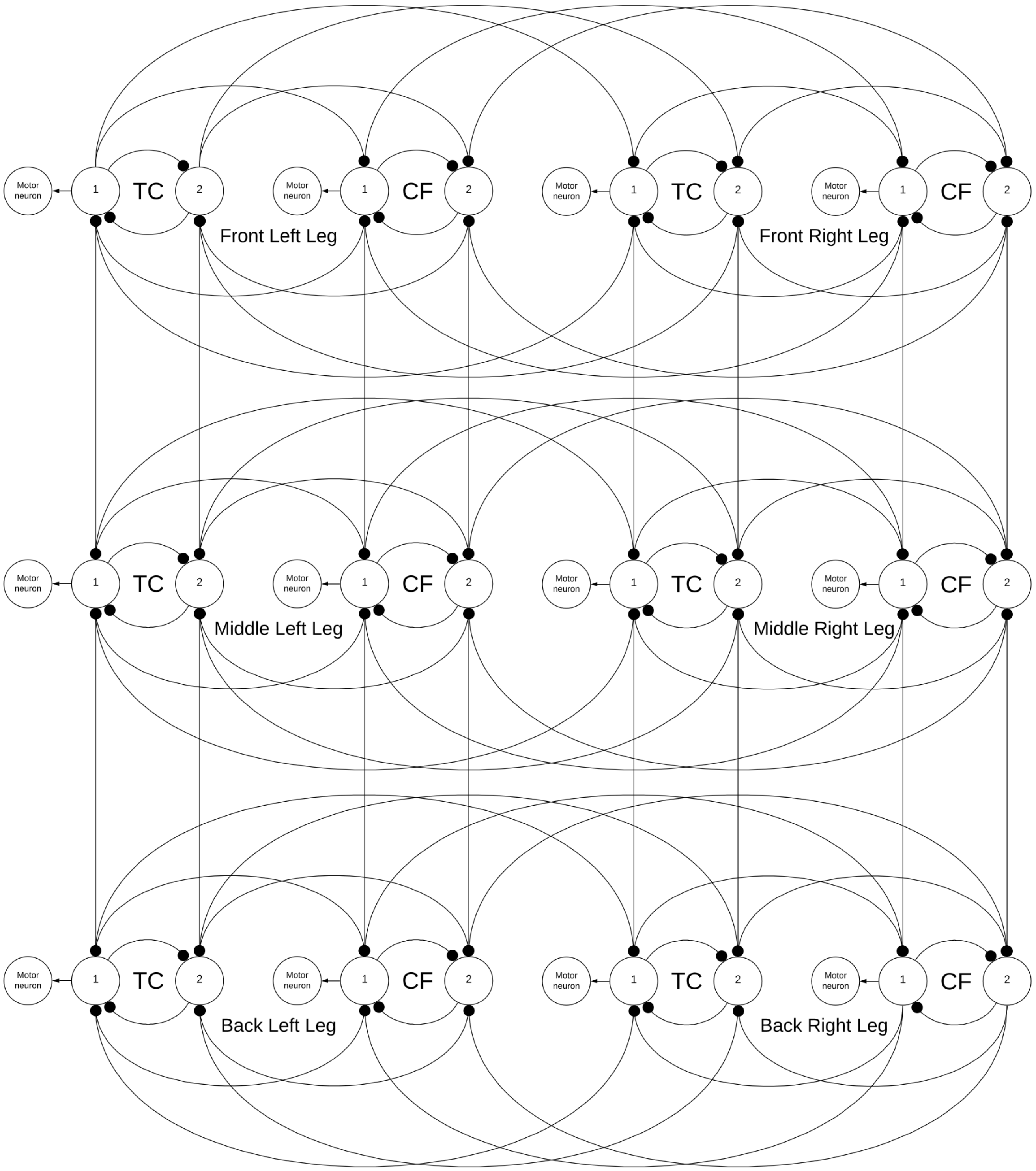

Figure S5. Complete diagram showing all connections between individual neuron populations in the distributed network. The inter-segmental mutual inhibition connections are shown as a single connection with inhibition on either end to simplify the diagram.
